# Antimicrobial Activity of Cinnamon, Tea Tree, and Thyme Essential Oils Against Pathogenic Bacteria Isolated from Tilapia (*Oreochromis* spp.) in Aquaculture Farms

**DOI:** 10.3390/molecules30132799

**Published:** 2025-06-28

**Authors:** Karen A. Terrazas-Pineda, Liliana Alamilla-Beltrán, Claudia Ariadna Acero-Ortega, Juan Antonio Damas-Espinoza, Georgina Calderón-Domínguez, Rosalva Mora-Escobedo, Vicente Vega-Sánchez, Fabián Ricardo Gómez-de Anda

**Affiliations:** 1 Departamento de Ingeniería Bioquímica, Escuela Nacional de Ciencias Biológicas, Instituto Politécnico Nacional, Unidad Profesional Adolfo López Mateos, Zacatenco, Av. Wilfrido Massieu 399, Col. Nueva Industrial Vallejo, Alcaldía Gustavo A. Madero, Ciudad de México CP 07738, Mexico; mvz.karenatp@gmail.com (K.A.T.-P.); jdamasantonio@gmail.com (J.A.D.-E.); gcalderon@ipn.mx (G.C.-D.); rmorae@ipn.mx (R.M.-E.); 2 ESDAI, Universidad Panamericana, Augusto Rodin 498, Ciudad de México CP 03920, Mexico; cacero@up.edu.mx; 3 Área Académica de Medicina Veterinaria y Zootecnia, Instituto de Ciencias Agropecuarias, Universidad Autónoma del Estado de Hidalgo, Avenida Universidad Km. 1, Ex hacienda Aquetzalpa, Tulancingo CP 43600, Hidalgo, Mexico; vicente_vega11156@uaeh.edu.mx (V.V.-S.); fabian_gomez9891@uaeh.edu.mx (F.R.G.-d.A.)

**Keywords:** *Vibrio*, *Aeromonas*, *Pseudomonas*, thymol, cinnamaldehyde, antibiotic resistance

## Abstract

Overexploitation has led to a rise in pathogenic bacteria within aquaculture, increasing reliance on antibiotics, and developing microorganism resistance. This situation underscores the need to explore alternatives with a reduced ecological impact. Metabolites derived from essential oils have demonstrated antimicrobial properties that can inhibit or diminish the activity of various microorganisms. In this study, the antimicrobial efficacy of cinnamon (*Cinnamomum zeylanicum)*, tea tree (*Melaleuca alternifolia*), and thyme (*Thymus vulgaris*) essential oils against pathogenic bacteria (*Aeromonas*, *Pseudomonas*, *Shewanella*, *Comamonas*, *Vibrio*, *Acinetobacter*, and *Empedobacter*) isolated from tilapia (*Oreochromis* spp.) brooded in Hidalgo State, Mexico, were investigated. Diffusion tests were conducted using discs infused with 12 different antibiotics and discs infused with essential oils at concentrations of 15, 10, and 5 μL each. Minimal inhibitory concentration tests were performed using a 96-well microplate format. All bacterial strains exhibited multi-resistance to various antibiotics; however, thyme and cinnamon effectively inhibited the tested bacteria at the lowest concentrations, while tea tree oil was the least effective. The findings suggest the potential incorporation of thyme and cinnamon as an alternative prevention to decrease the use of antibiotic treatment.

## 1. Introduction

Fish play a crucial role worldwide as a source of nutrients. Their high protein and fatty acid content make them a healthy alternative, shown to help reduce the inflammatory process [1], contributing to their increasing popularity; moreover, they provide livelihood and income to more than 100 million people worldwide [2]. Tilapia (*Oreochromis* spp.) is a promising species since it can grow in many aquaculture systems, reproduce in a wide range of environmental conditions, and is highly tolerant to handling practices [3]. However, intensification in farms and some practices lead to outbreaks of diseases—some caused by pathogens already present, elevating the mortality rate—and economic losses [4]. *Aeromonas* spp., *Streptococcus* spp., *Edwarsiella* spp., *Staphylococcus* spp., *Pseudomonas* spp., *Vibrio* spp., and *Plesiomonas* spp. are some of the genera pointed out as causes of diseases [5,6,7]. As a result, antibiotics are used, either administered in food or directly in water; however, residual water can drag residues of the drugs used, due to the lack of proper infrastructure in the management of water, carrying it to aquatic environments where antimicrobial residues can reach other areas of production [8]. Moreover, fish fillets can also contain antimicrobial residues and cause negative effects, such as allergies and gastrointestinal microbiota disturbances [9]. The conditions above pave the way for bacteria to acquire antibiotic resistance, representing a current threat to public health. Many countries are working on strategies to reduce antimicrobial use and their free sale [10]; however, antimicrobials utilized in Mexico are not specifically designed for aquatic environments, and some are even banned for use in animals intended for human consumption [11].

Plants and their metabolites exhibit potential for application in aquaculture due to their diverse range of bioactive compounds. These compounds have demonstrated various beneficial functions, such as appetite stimulation, growth promotion, and stress reduction, among others [12]. Also, there is increasing evidence of their antimicrobial properties against multiple bacteria, yeast, and parasites [13,14]. Essential oils (EOs) are products from different parts of plants (flowers, leaves, roots, seeds, fruits, wood, and bark) extracted by steam distillation, hydro diffusion, or pressure [15]. EOs are aromatic, light, volatile, and insoluble in water, and their principal components are flavonoids, terpenes, tannins, alkaloids, and coumarins [16]. Their use as treatments in diverse species has been supported as an alternative because they are reliable, biodegradable, have a low impact on the environment, have very few side effects, are less likely to cause the onset of antibiotic resistance, and their supplementation in diets can improve food intake and the immune system and stimulate growth of beneficial bacteria [17,18,19,20].

Cinnamon (*Cinnamomum zeylanicum*) is a highly appreciated spice worldwide. Leaves and stems of cinnamon can be used to treat diseases, and its essential oil contains 1,8-Cineole, caryophyllene oxide, eugenol, camphene, cinnamaldehyde, linalool, α-pinene, and cinnamyl acetate, among others, linked to antimicrobial activity [17]. Thyme (*Thymus vulgaris)* is a member of the *Lamiaceae* family, and its essential oil contains monoterpene phenols, such as thymol, carvacrol, p-cymene, γ-terminen, limonene, borneol, and linalool [18]. Antibacterial activity is linked to the presence of thymol and carvacrol, causing bacteriostatic effects against Gram-positive and negative bacteria [21]. *Melaleuca alternifolia*, or tea tee, as it is commonly known, is an Australian plant; its essential oil has been widely distributed in Asia, Europe, and Australia and is used in personal health items because of its antimicrobial properties, attributed to its bioactive compounds (p-cymene, terpinen-4-ol, terpinolene, 1, 8-cineole, α-pinene, γ-terpinene) [22,23,24].

Essential oils (EOs) exert a multifaceted antimicrobial action that distinguishes them from conventional antibiotics. While the latter act on specific molecular targets, EOs simultaneously affect multiple cellular structures, such as the membrane, enzymes, DNA, and energy metabolism [20]. Their mechanisms include disruption of the cell membrane, interference with respiration and protein synthesis, and destabilization of genetic material. This non-specific action hinders the development of bacterial resistance, promotes efficacy against multi-resistant strains, and enhances synergistic effects between its components [21].

Due to the urgent need to develop new strategies to treat or lessen the use of antibiotics in aquaculture and their growing resistance to antimicrobials, this study aimed to evaluate and compare antibiotics and essential oils (EOs) against previously isolated strains of bacteria from tilapia as a possible alternative to reduce the effects of pathogenic bacteria. This study was designed as a preliminary in vitro analysis focusing on the basic antibacterial activity of the selected essential oils (EOs) against previously characterized resistant strains

## 2. Results

### 2.1. Antibiogram

High resistance rates were observed in almost all isolates tested (Table 1), and only two strains, *Acinetobacter Iwoffii* and *Empedobacter falsenii* (16%) were intermediate or sensitive to the action of antibiotics. All isolates (100%) were resistant to aminoglycosides (amikacin, gentamicin, and netilmicin), while only the *Empedobacter falsenii* (8.33%) strain was sensitive to beta-lactams (ampicillin and carbenicillin), cephalosporins (cephalothin and cefotaxime), quinolones (ciprofloxacin and norfloxacin), nitrofurans/phenicols (norfloxacin and chloramphenicol), and sulfonamides (sulfamethoxazole).

The antibiogram results revealed that the strains exhibited multidrug resistance (MDR), with MAR (Multiple Antibiotic Resistance) indices ranging from 0.36 to 1.00. Notably, *Aeromonas* spp., *Pseudomonas anguilliseptica*, *Shewanella putrefaciens*, *Comamonas thiooxydans*, and *Vibrio cholerae* showed resistance to more than three antibiotic classes, confirming their MDR status (Table 1). All *Aeromonas* strains showed resistance to all antibiotics, as well as *P. anguilliseptica*, *S. putrefaciens*, *C. thiooxydans*, and *V. cholerae*, while *A. Iwoffii* showed resistance to all antibiotics except for sulfamethoxazole. *E. falsenii* was resistant to amikacin, gentamycin, ciprofloxacin, sulfamethoxazole, and netilmicin, but proved sensitive to ampicillin, carbenicillin, cephalothin, cefotaxime, norfloxacin, chloramphenicol, and norfloxacin. We found a range of 0.50 to 1.00, which can be a clue to the use of antibiotics in the farming systems where the strains were isolated [25].

### 2.2. Essential Oils (EOs) Disc Diffusion Test (DDt)

**Figure 1:**
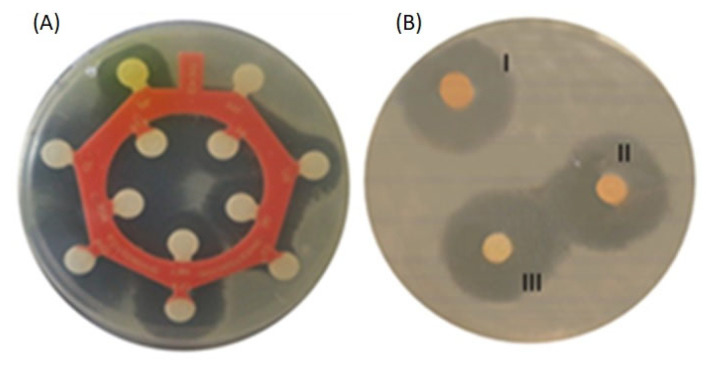
Comparative results of *Aeromonas veronii* from antibiogram and disk diffusion testing of essential oils. (**A**) Antibiogram. (**B**) Disk diffusion of cinnamon essential oil: (I) corresponds to the disk impregnated with 15 µL, (II) to the disk impregnated with 10 µL, and (III) to the disk impregnated with 5 µL.

The EO that had the biggest inhibition zone was thyme, while tea tree was the least effective because it was significantly lower in all tests. The bacteria with the lower results were *V. cholerae* and *A. Iwoffii*. The results of the measurement of inhibition halos and their means are shown in Table 2. The inhibition zones observed in CEO ranged from 5.33 to 18.00 mm; 0.93 to 13.67 mm was the range for TTEO; and 4.67 to 19.67 mm was the range for TEO. Cinnamon showed higher mean inhibition halos in *Aeromonas* sp., *Aeromonas veronii*, *Aeromonas sobria*, *Shewanella*, and *Comamonas thiooxydans;* in contrast, *Aeromonas dhakensis*, *Pseudomonas*, and *Empedobacter* showed higher mean inhibition halos for thyme DDt. *Vibrio cholerae* and *Acinetobacter Iwoffii* shared similar results using TEO and CEO. Comparative images of DDt and antibiogram testing are shown in Figure 1.

**Figure 2:**
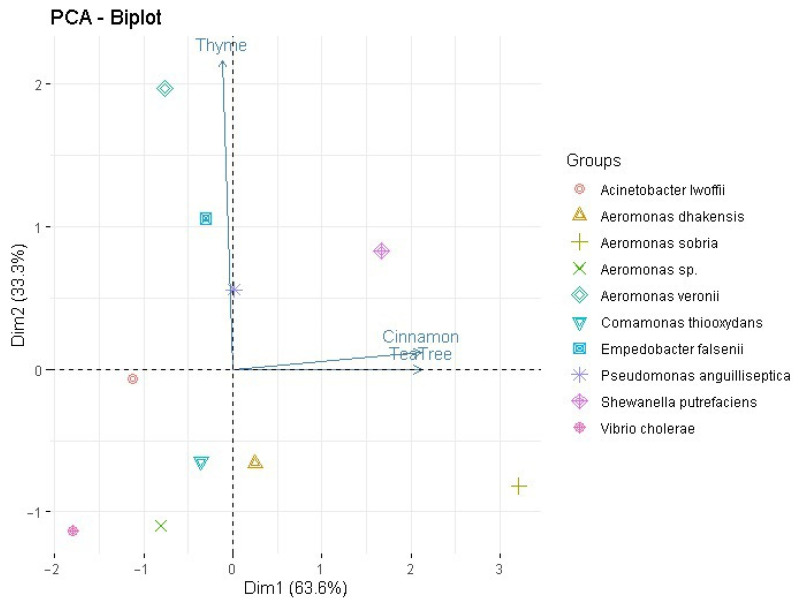
Principal Component Analysis (PCA) shows distinct differences in the antibacterial activity of cinnamon, thyme, and tea tree essential oils against various bacterial species. The first two principal components account for 96.9% of the total variability in the data: Dim1 contributes 63.6% and Dim2 33.3%.

Principal Component Analysis (PCA) was performed, which showed distinct differences in the antibacterial activity of cinnamon, thyme, and tea tree essential oils against various bacterial species (Figure 2).

*Aeromonas veronii*, *Shewanella putrefaciens*, and *Comamonas thiooxydans* are highly associated with thyme essential oil, suggesting that this compound significantly impacts their antibacterial activity. *Pseudomonas anguilliseptica*, *Enterobacter falsenii*, and *Acinetobacter lwoffii* are located near the cinnamon vector, suggesting that the compounds in this essential oil may be key in inhibiting bacterial growth. *Vibrio cholerae* and *Aeromonas sobria* are closer to the tea tree oil vector, suggesting that compounds such as terpinene-4-ol and α-terpineol may play a key role in the antibacterial activity of these species.

### 2.3. Minimal Inhibitory Concentrations (MICs)

All strains tested (100%) showed sensitivity to thyme at the lowest concentration (0.12); similar results were found in cinnamon, where the sensitivity ranges were 0.12 to 2%. Tea tree was the least effective, with the widest range, at 0.12 to 4%.

**Figure 3:**
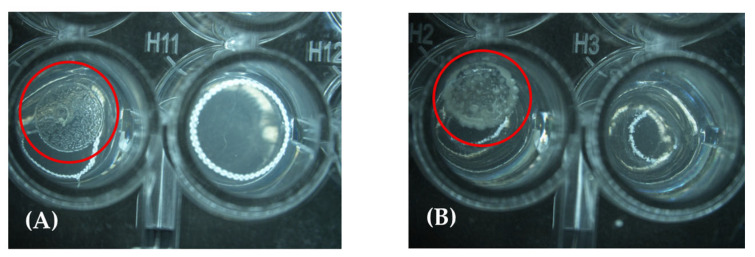
Comparative images of two strains evaluated in the MIC assay at the lowest concentration (0.12% *v*/*v*) against TTEO; (**A**) corresponds to *Vibrio cholerae* and (**B**) to *Empedobacter falsenii*.

All *Aeromonas* strains were sensitive to the lowest concentration of thyme, followed by cinnamon (Table 3); only two strains needed a higher concentration of 1% to inhibit the development. The range in the tea tree case was even higher, from 1.0 to 4.0%. The lowest concentration of TEO inhibited *Pseudomonas* (Figure 3), while cinnamon and tea tree were at 0.5%, like *Shewanella*, where a higher concentration of cinnamon and tea tree EOs was needed (2%). *Comamonas* strain was inhibited by cinnamon at 1% and by tea tree at 0.5%; *Vibrio* needed the same concentration of cinnamon oil, while 2% was used for tea tree. *Acinetobacter* needed the same amount of cinnamon and tea tree EOs (1%). *Empedobacter* had no development at (0.12%) for thyme and tea tree, while 0.25% was needed for cinnamon.

## 3. Discussion

The composition of bioactive molecules varies depending on several plant characteristics such as location, age, climate, temperature, and the part of the plant sampled, with the collection and obtaining of essential oils also being important [23]. Many can be found in different EOs; Table 4 summarizes the main components in each EO used and some molecules reported in the manufacturer’s datasheet (doTERRA Holdings, LLC. Pleasant Grove, UT, USA). Tea tree essential oil (TTEO) and thyme essential oil (TEO) both showed the presence of γ-terpinene and p-cymene; in TEO and cinnamon essential oil (CEO), linalool was found; and TTEO and CEO shared the presence of α-pinene. P-cymene is a common ingredient in food flavoring and in the production of fungicides and pesticides [24]. Gamma-terpinene is the precursor of thymol and p-cymene and possesses low toxicity in animal models; along with its isomer, α-terpinene shares an antioxidative effect [25]. The monoterpene α-pinene displays antioxidant, antifungal, antibacterial, and anti-inflammatory activity [26]. Linalool is a monoterpene that represents up to 90.6% of the oil constituents of over 200 aromatic plant species of different families [27]; combinations with florfenicol or oxytetracycline against *A. hydrophila* demonstrated a synergic effect [28]. Terpinen-4-ol, the main active constituent of tea tree oil, possesses antiseptic, antioxidative, and sedative activity, along with α-terpineol, which has been pointed to as responsible for its antimicrobial activity [29]. Its addition in diets containing tilapia proved to increase nutrient absorption and growth rates, and even helping in the immune response against bacteria like *Aeromonas* [24,25,26,27,28,29,30]. The favorable hydrophobic/hydrophilic character of terpinen-4-ol is thought to be the basis for its antimicrobial activity [31]. Thymol, the main component in TEO, is a monoterpenoid phenolic compound that exists naturally along with its isomer carvacrol; both exhibit a distinct set of biological activities, including antioxidant, antitumor, antibacterial, antifungal, anesthetic, and insecticidal properties [27]. Trans-cinnamaldehyde is a natural product from cinnamon oils; its cis counterpart, cinnamaldehyde, cannot be found in nature; thus, the word “trans” is usually omitted. Its multiple mechanisms of action against bacteria have been studied because of its ability to cause alterations in membrane cells and inhibition of cell division, motility, biofilm formation, and quorum sensing [32]. The carbonyl group of cinnamaldehyde is considered responsible for antimicrobial action by binding to cellular proteins, preventing them from functioning properly [33]. Eugenol can be found in many EOs, possesses bacteriostatic activity, and has a major spectrum of action against Gram-negative bacteria [34].

Bioactive constituents of EOs have many mechanisms of action. Gram-positive bacteria are more sensitive to plant extracts than Gram-negative bacteria because the cell wall of Gram-negative bacteria is more complex due to the outer membrane and lipopolysaccharides (LPS), conferring greater resistance to hydrophobic compounds [32]. This could be a disadvantage because most bacteria that affect fish and those used in our test are Gram-negative. The mechanisms of action against these bacteria are described as binding to the surface of bacterial cells and subsequently disrupting the integrity of the cell membrane’s phospholipid bilayer; inhibition of ATP; inhibition of peptidoglycan synthesis or denaturation of proteins; and disturbing the structural integrity of the cell membrane, hence exerting detrimental effects on cellular metabolism, and ultimately leading to cell death [35]. However, the effectiveness of antimicrobial activity has been attributed to the interactions between major and minor bioactive components in each oil [36].

Antimicrobial resistance against EOs is a concern, but it can be considered a minor threat since the multi-component nature of EOs may reduce the potential for resistance to occur spontaneously, and it would require multiple simultaneous mutations to overcome each component’s antimicrobial actions [29]. In some trials, repetition up to 50 times did not show development of resistance [37,38].

There have been different strategies in aquaculture to incorporate the benefits of bioactive compounds present in plants. Some involve the supplementation in diets of extracts like garlic, which, when tested against *A. hydrophila*, proved to reduce mortalities [39]. Öntas et al. [40] added sweet orange peel (a cheap source of orange essential oil) to fish feed, and it conferred resistance to *Streptococcus iniae* infection in Mozambique tilapia. Sönmez et al. [41] observed positive effects from the addition of thyme and sage EOs to the diet of rainbow trout.

As pointed out before, the antimicrobial activity of EOs is effective because of the action of their combined constituents. CEO has proven to inhibit a wide range of bacteria. Anandhi et al. [42] evaluated its performance against bacteria isolated from wounds. It showed high antibacterial activity attributed to cinnamaldehyde, benzoic acid, benzaldehyde, and cinnamic acid. Sousa et al. [43] observed synergistic effects in the combination of carvacrol and p-cymene, caused by the interaction of p-cymene with the lipidic membrane of cells, followed by the expansion of the membrane, which facilitated and increased the transportation of carvacrol into the cells. The effectiveness of TEO in the MIC test could be attributed to this type of interaction, since carvacrol and p-cymene can be found among the bioactive components. Our results with respect to the DDt for CEO differ slightly from those observed by Rattanachaikunsopon and Phumkhachorn [33]. Their assays resulted in inhibition halos of 21.5 mm using 30 µL of CEO, while our results showed 16.67 mm at 15 and 10 µL. Their analysis of CEO showed a relatively higher concentration of cinnamaldehyde (90.24%), along with the presence of limonene (2.42%), linalool (1.16%), α-terpineol (0.84%), and cinnamyl acetate (2.03%), hinting that, while cinnamaldehyde has elevated antimicrobial activity, its synergy with other components like cinnamyl acetate or linalool can affect its effectiveness. Hudecová [44] observed bigger inhibition zones for *Aeromonas veronii* isolated from trout for TEO (43.0mm) and TTEO (39.33 mm) at a dose of 10 µL. Similar results were observed by Kacániová et al. [45] when evaluating CEO and TEO against several strains of *Pseudomonas* isolated from fish, with results ranging from 15.00 mm to 9.67 mm, respectively.

Cinnamon and thyme EOs showed better performance in comparison with TTEO, with the latter seeming to need to be used at a higher concentration. Mumu and Hossain [46] observed this effect in their evaluation of EOs from eucalypt, lemongrass, and tea tree at 50 µL against pathogenic bacteria, where TTEO was the most effective and had the biggest inhibition zones (36.33 mm against *A. hydrophila*). The MICs we observed in *Shewanella putrefaciens* required a bigger dose in CEO than the results declared by Lyu et al. [47]. Golus et al. [48] found MICs of 0.06 (%*v*/*v*) in CEO and 0.25 (%*v*/*v*) in TTEO tested against *Pseudomonas aeruginosa*, differing from the 0.5 (%*v*/*v*) we obtained when testing against *Pseudomonas anguilliseptica*. Nonetheless, many authors suggest the differences could be attributed to the testing methods, variability of EOs, and microbial strains.

In our study, CEO and TEO showed the best results in terms of DDt in the majority of the microorganisms tested. TEO inhibited all strains at 0.12 (%*v*/*v*), and all strains were sensitive to the action of EOs. However, the smallest inhibition zones were observed in *Vibrio cholera* and *Acinetobacter Iwoffii*, the first one a major microorganism of concern and the second one an emerging pathogen; hence, further studies should be carried out to explore other EOs. *Aeromonas* strains emerge as pathogens of both human and animal health, but our results showed a good effect with respect to CEO against these bacteria. The results suggest that thyme strongly affects *Aeromonas veronii* and *Shewanella putrefaciens*, while cinnamon influences *Pseudomonas anguilliseptica* and *Enterobacter falsenii*. Conversely, tea tree oil appears to be more effective against *Vibrio cholerae* and *Aeromonas sobria*. These findings align with previous studies and demonstrate the selective antibacterial activities of these essential oils against specific bacterial strains. The variations between studies indicate the need for standardized procedures for substances whose composition is a mix of complex molecules, like EOs, and for emerging microorganisms, as no guidelines are established for these. As we have shown, there is a growing resistance to the effects of antibiotics in aquaculture. Nonetheless, our results on EOs showed that they can be used as future alternatives with respect to improving and treating infections caused by bacteria in tilapia breeding. Moreover, new routes of administration could be explored.

The area of the Mezquital Valley has a growing community of tilapia producers (60.22% of the total state aquaculture production) [49], who receive a mixture of urban, industrial, and rain wastewater from the City of Mexico, which is contaminated with diverse drugs [50]. Lesser [51] and Garduño-Jiménez et al. [52] found carbamazepine, erythromycin, flumequine, ofloxacin, ormetoprim, oxacillin, oxytetracycline, sulfadiazine, sulfadimethoxine, sulfamethazine, sulfamethoxazole, and trimethoprim in Mezquital Valley. This is a current problem for aquaculture, since heavily polluted waters can also have teratogenic effects in fish embryos [53].

All the strains evaluated are Gram-negative bacilli of different groups with wide environmental distribution and emerging antimicrobial activity. *Aeromonas* spp. are associated with aquatic environments such as lakes, rivers, ponds, seas, estuaries, drinking water, sediments, and wastewater [54]. They have also been isolated in meat, raw milk, poultry, fish, shellfish, and vegetables [55]. They are an opportunistic pathogen of domestic animals, amphibians, reptiles, fish, and even humans [56]. Infected fish develop lesions like ulcers in the skin, ascites, hemorrhagic septicemia, furunculosis, and disorders in the gastrointestinal tract, liver, and kidney [57]. *Pseudomonas anguilliseptica*, an opportunistic fish pathogen of salt and fresh water, causes septicemia, petechial bleeding lesions, and subcutaneous edema, among others, finally causing death between 1 and 2 days after infection [58]. *Vibrio cholerae* can be isolated from aquatic environments, especially in hot months, and has been pointed out as an occasional pathogen of fish and shrimp, among other aquatic animals [59]. Consumption of seafood or raw or undercooked fillets causes deep dehydration, dysentery, and, in some cases, extraintestinal diseases such as meningitis or septicemia [60]. *Shewanella putrefaciens* has great relevance in aquaculture and the food industry, due to the conditions it causes on fish such as skin lesions and ulcers, while provoking mortalities of up to 85% [61]. In addition, it can survive refrigeration temperatures, causing rotting meat of aquaculture, bovine, or poultry origin [62]. *Acinetobacter Iwoffii* is an emerging aquaculture pathogen that causes lesions such as skin hemorrhages, enteritis, and ascites in bullfrog, catfish, and common carp [63]. *Comamonas thiooxydans* belongs to *the Comamonas* genus, whose members have been isolated from sites heavily contaminated with complex organic compounds and heavy metals, and increasingly from clinical environments, thus regarded as a rare opportunistic pathogen [64]. *Empedobacter falsenii*, formerly known as *Wautersiella falsenii*, was first described in 2006; it is not a common cause of human infection [65].

Treatments in aquaculture usually mean antibiotics, but as has been heavily implied in this study, constant exposure to these substances has led to antimicrobial resistance. Our results show a heavy contrast to those reported worldwide. Outbreaks of *P. anguilliseptica* resistant to ampicillin, kanamycin, penicillin, and colistin have been reported [66], similar to our results; thus, our isolate was resistant to aminoglycosides, an antibiotic group where ampicillin, kanamycin, amikacin, and gentamicin belong. Hassan et al. [67] reported strains isolated from fish farms sensitive to norfloxacin, ciprofloxacin, and gentamycin, contrary to our results, where the strain evaluated showed resistance to those antimicrobials. With *Aeromonas* spp. and *Vibrio cholerae*, there are alarming reports of antibiotic resistance in species like tilapia, koi carp (*Cyprinus carpio koi*), and catfish (*Ictalurus punctatus*) [68]. Elgendy et al. [69] found strains resistant to ampicillin and amoxicillin but sensitive to ciprofloxacin and trimethoprim/sulfamethoxazole in farms in Egypt with mortality episodes suspected as a result of improper use of antibiotics; in our results, *Aeromonas* and *Vibrio* were resistant to ciprofloxacin and sulfamethoxazole. Contrary to our results, Jiang et al. [70] found strains of *Shewanella* isolated from largemouth bass (*Micropterus salmoides*) susceptible to cefotaxime, sulfamethoxazole, gentamicin, ampicillin, and norfloxacin. Kozińska [71] also reported an *A. Iwoffi* strain resistant to ampicillin, amoxicillin, and cephalothin, intermediate in terms of cefuroxime and oxytetracycline and sensitive to enrofloxacin, flumequime, norfloxacin, gentamycin, and sulfamethoxazole/trimethoprim. Zhang et al. [72] isolated strains resistant to florfenicol, sulfadiazinum, penicillin, and tetracycline but sensitive to neomycin sulfate, minocycline, doxycycline, polymyxin B, amikacin, gentamycin, and ampicillin. Cao et al. [73] found strains with similar resistance to ampicillin, cefotaxime, and gentamicin, but moderately sensitive to ciprofloxacin and norfloxacin. *Comamonas* spp. can be resistant to many antibiotic families, including β-lactams (penicillins, cephalosporins, carbapenems), with treatments based upon in vitro assays testing isolates [74]. Guo [75] reported a case of isolation of *C. thiooxydans* from urine in a coinfection with *E. coli* from a patient with a urinary tract infection; the strain proved resistant to aztreonam, fluoroquinolones, and aminoglycosides. However, some clinical cases report strains resistant to aztreonam, ampicillin–sulbactam, cephalothin, meropenem, and colistin; intermediate with respect to piperacillin–tazobactam, cefotaxime, ceftazidime, imipenem, and ciprofloxacin; and susceptible to cefepime, amikacin, gentamicin, and trimethoprim–sulfamethoxazole [67,76], but our study demonstrated *C. thiooxydans* resistant to gentamicin and sulfamethoxazole.

## 4. Materials and Methods

### 4.1. Essential Oils

The essential oils (EOs) utilized in this research included cinnamon (*Cinnamomum zeylanicum*), tea tree (*Melaleuca alternifolia)*, and thyme (*Thymus vulgaris*) sourced from doTERRA (doTERRA Holdings, LLC; 2021). The analysis for each oil was provided by the manufacturer (Gas Chromatography). The major components of each EO are presented in Table 4. The essential oils were used in their pure form for the disk diffusion method. When conducting Minimal Inhibitory Concentration (MIC) testing, the essential oils were diluted in DMSO (dimethyl sulfoxide; Sigma Aldrich, Saint Louis, MO, USA) at a concentration of 2.0–1.0% (*v*/*v*) and added in molten MH Agar (Mueller Hinton, MCD. LAB, Tlalnepantla, México)

### 4.2. Bacterial Strains

The bacterial strains used in this study were previously isolated from various municipalities in Hidalgo State, Mexico, during the month of August 2021 and identified by PCR for *gcaT*, *rpoD*, and *16s rRNA* genes following the methodology fully described by Zepeda et al. [77]. Details about the strains are provided in Table 5. Subcultures were prepared from isolates previously preserved in Brain Heart broth (MCD Lab, Mexico) and stored in vials at −20 °C until required [78]. To generate fresh cultures before each test, isolates were grown on Mueller–Hinton agar at 35 °C. All bacterial isolates used in this study were subjected to antibiotic susceptibility testing following EUCAST guidelines. The antibiogram results revealed that the strains exhibited multidrug resistance (MDR), with MAR (Multiple Antibiotic Resistance). The identity of each bacterial strain was confirmed by PCR and Sanger sequencing. Specific genes used for molecular identification included gcaT and rpoD for Aeromonas and 16S rRNA for all strains. The sequences were compared against the NCBI GenBank database using the online version of BLAST (https://blast.ncbi.nlm.nih.gov/Blast.cgi) (accessed on August 2022), and all isolates showed >98% identity with reference sequences. The GenBank accession numbers for each strain are listed in Table 5.

### 4.3. Inoculum Preparation

The inoculum was prepared using the CLSI (Clinical & Laboratory Standards Institute) direct colony suspension method. Bacterial colonies following 18–24 h of growth were suspended in Mueller–Hinton broth and adjusted to a turbidity of 0.5 MacFarland standard, equivalent to 1–2 × 10^8^ colony forming units (CFUs) mL^−1^ [79]. For the Minimal Inhibitory Concentration test (MIC), the inoculums were diluted 1:10 in Mueller–Hinton broth to achieve a concentration of 10^7^ CFUs mL^−1^. Then, 2 µL was applied to the agar surface of each well in the microplate to accomplish a final inoculum density of approximately 10^4^ CFUs per well [49]. All bacterial suspensions were used within 15 min after preparation.

### 4.4. Antibiogram

The antibiogram was used to evaluate the sensitivity of the isolates to several antibiotics, and it was performed according to the CLSI M02-A11 [79]. The inoculum was prepared as described above. A sterile swab was introduced into the inoculum and then rotated against the tube wall to eliminate excess broth. Next, the swab was used to strike the surface of Mueller–Hinton agar plates and left to dry for 15 min before placing antibiotic discs (PT-35 Multibac I.D. for Gram-negative bacteria, Mexico). The antibiotics used were ampicillin (AM; 10 mcg), amikacin (AK; 30 mcg), carbenicillin (CB; 100 mcg), gentamicin (GE; 10 mcg), cephalothin (CF; 30 mcg), cefotaxime (CFX; 30 mcg), ciprofloxacin (CPF; 5 mcg), norfloxacin (NOF; 10 mcg), chloramphenicol (CL; 30 mcg), sulfamethoxazole (STX; 25 mcg), nitrofurantoin (NF; 300 mcg), and netilmicin (NET; 30 mcg). Antibiotic discs were left on the surface of plates and incubated at 35 °C for 18 h. Afterward, plates were placed against a dark background and photographed, and the diameter of the inhibition halos was measured. According to the standards proposed by CLSI [79] and EUCAST [80], bacteria were classified as Susceptible (S), Susceptible, increased exposure (I), and Resistant (R); for organisms without guidelines, interpretative criteria for similar antimicrobial or organism combinations were used. The MAR index was used, which is the number of antibiotics to which the isolate was resistant divided by the number of antibiotics to which the isolate was exposed [81]

### 4.5. Essential Oils (EOs) Disc Diffusion Test (DDt)

The CLSI protocol for disc diffusion was adapted to assess the efficacy of EOs against pathogenic bacteria. A similar process to the one mentioned above was performed to inoculate Mueller–Hinton plates with inoculum, previously prepared and adjusted to a turbidity of 0.5 MacFarland standard, equivalent to 1–2 × 10^8^ colony forming units (CFUs) mL^−1^, using sterilized filter paper discs (6 mm) impregnated with 15, 10, and 5 µL of each oil. These discs were placed on the Petri dishes’ surface and incubated at 35 °C for 24 h. The test was conducted in triplicate, and the results were analyzed using one-factor ANOVA with the Tukey method (means *p* < 0.05 are significantly different). Additionally, a Principal Component Analysis (PCA) was performed to evaluate differences in the antibacterial activity of cinnamon, thyme, and tea tree essential oils against various bacterial species

### 4.6. Determination of Minimum Inhibitory Concentration (MIC)

**Figure 4:**
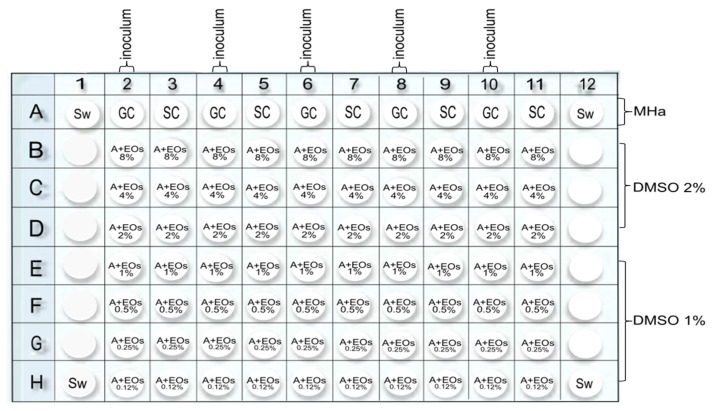
Minimal Inhibition Concentration (MIC) plate test distribution. Sterile water (Sw) is placed in each corner; Mueller–Hinton agar (MHa) without EO is placed in the top row as a first Grow Control (GC) and Sterile Control (SC). Two columns are used for each pathogen, with paired numbers inoculated and unpaired numbers left as a second sterile control. The concentration of EOs has a descending range of 8.0–0.12% (*v*/*v*).

The MIC of EOs was evaluated following the methodology proposed by Golus et al. [48]. Sterile, flat-bottomed 96-well plates (Corning) were utilized. All oils were used in the 8.0–0.125% (*v*/*v*) range. These were poured into test tubes to which 2% (*v*/*v*) DMSO was added, ensuring the final volume did not exceed this concentration; following this, melted Mueller–Hinton agar was added, achieving 100 µL for each well. The tubes were subjected to vortexing and placed in a digital reactor (Hach DRB200) at 50 °C until use. The melted Mueller–Hinton agar was subsequently added to achieve volumes of 100 µL per well in all plates, except for the first file, where only molten Mueller–Hinton agar was left. The plates were left at room temperature until solidified, and then the bacterial suspensions previously prepared were inoculated in the plate, as shown in Figure 4. For each bacterium, two columns were used: inoculum wells (2, 4, 6, 8, 10) and uncultured wells (3, 5, 7, 9, 11). A plate was used for every EO. The MIC was defined as the lowest dilution with no visible growth of the microorganism (absence of turbidity). Incubation at 35 °C may lead to excessive evaporation, potentially causing agar well desiccation and compromising the hydration status of the culture. Supplementing with sterile water could mitigate this issue, particularly in wells exhibiting rapid evaporation rates, which may otherwise adversely impact bacterial growth dynamics.

## 5. Conclusions

This study presents a promising alternative for enhancing fish health, specifically in tilapia reared in aquaculture farms, considering findings regarding potential antibiotic resistance in pathogens isolated from these fish. The observed improvements in fish health can be attributed to the antimicrobial properties of essential oils, particularly in cinnamon and thyme, which exhibit effectiveness against microorganisms such as *Vibrio cholerae* and *Aeromonas*. These essential oils show potential as natural agents for controlling bacterial proliferation, offering a viable alternative to the unrestricted use of antibiotics in farming practices. Based on these results, there is an opportunity for further, more in-depth studies across additional farms to explore various applications and methods of administration, as well as an evaluation of the effectiveness of EOs against different fish species.

## Figures and Tables

**Table 1:** Antibiogram results of bacteria tested against several antibiotics.

Microorganism	Resistant to	MAR Index
*Aeromonas* sp.	AM, AK, CB, GE, CF, CFX, CPF, NOF, CL, STX, NF, NET	1.00
*Aeromonas* *dhakensis*	AM, AK, CB, GE, CF, CFX, CPF, NOF, CL, STX, NF, NET	1.00
*Aeromonas veronii*	AM, AK, CB, GE, CF, CFX, CPF, NOF, CL, STX, NF, NET	1.00
*Aeromonas veronii*	AM, AK, CB, GE, CF, CFX, CPF, NOF, CL, STX, NF, NET	1.00
*Aeromonas veronii*	AM, AK, CB, GE, CF, CFX, CPF, NOF, CL, STX, NF, NET	1.00
*Aeromonas sobria*	AM, AK, CB, GE, CF, CFX, CPF, NOF, CL, STX, NF, NET	1.00
*Pseudomonas anguilliseptica*	AM, AK, CB, GE, CF, CFX, CPF, NOF, CL, STX, NF, NET	1.00
*Shewanella putrefaciens*	AM, AK, CB, GE, CF, CFX, CPF, NOF, CL, STX, NF, NET	1.00
*Comamonas thiooxydans*	AM, AK, CB, GE, CF, CFX, CPF, NOF, CL, STX, NF, NET	1.00
*Vibrio cholerae*	AM, AK, CB, GE, CF, CFX, CPF, NOF, CL, STX, NF, NET	1.00
*Acinetobacter Iwoffii*	AM, AK, CB, GE, CF, CFX, CPF, NOF, CL, NF, NET	0.83
*Empedobacter falsenii*	AK, GE, CPF, STX, NET	0.50

Antibiotics: AM, ampicillin; AK, amikacin; CB, carbenicillin; GE, gentamicin; CF, cephalothin; CFX, cefotaxime; CPF, ciprofloxacin; NOF, norfloxacin; CL, chloramphenicol; STX, sulfamethoxazole; NF, nitrofurantoin; and NET, netilmicin.

**Table 2:** Inhibition halos of bacteria tested against EOs in the disk diffusion test.

Microorganisms	Vol EOs (µL)	Cinnamon	Thyme	Tea tree
*Aeromonas* sp.	5 10 15	7.33 ± 0.57 ^Aa^ **8.33 ± 1.15 **^ABc^ 9.67 ± 0.57 ^Be^	**5.33 ± 1.55** ^Cab^ 6.67 ± 1.55 ^Ccd^ 8.67 ± 2.31 ^Ce^	3.33 ± 0.57 ^Db^ 4.67 ± 0.57 ^Dd^ **8.00 ± 1.00** ^Ee^
*Aeromonas* *dhakensis*	5 10 15	**9.33 ± 0.57 **^Ff^ 9.67 ± 0.57 ^Fg^ 10.67 ± 0.57 ^Fh^	**6.67 ± 1.15** ^Gf^ 8.33 ± 2.08 ^Gg^ 11.00 ± 2.65 ^Gh^	**5.67 ± 2.89** ^Hf^ 6.67 ± 2.08 ^Hg^ 10.33 ± 2.08 ^Hh^
*Aeromonas veronii*	5 10 15	**9.00 ± 3.46** ^Iij^ 9.33 ± 3.21 ^Ikl^ 10.67 ± 2.89 ^Imn^	**15.33 ± 4.16 **^Ji^ 16.00 ± 4.00 ^Jk^ 19.67 ± 7.51 ^Jm^	2.67 ± 0.57 ^Kj^ 4.67 ± 0.57 ^Ll^ **6.33 ± 0.57** ^Mn^
*Aeromonas veronii*	5 10 15	**10.00 ± 0.57 ^Nñ^** 10.67 ± 0.57 ^Ño^ 14.67 ± 0.57 ^Ñp^	**4.67 ± 1.5 ^Oñ^** 5.00 ± 1.73 ^Oo^ 6.33 ± 2.31 ^Oq^	**6.67 ± 2.36 ^Pñ^** 7.33 ± 4.04 ^Po^ 5.17 ± 2.36 ^Pq^
*Aeromonas veronii*	5 10 15	**9.67 ± 1.52 ^Qr^** 10.67 ± 0.57 ^Qt^ 13.33 ± 2.31 ^Qu^	**5.00 ± 1.00 ^Rrs^** 7.00 ± 2.65 ^Rt^ 7.67 ± 2.52 ^Ruv^	**3.00 ± 3.61 ^Ss^** 5.33 ± 3.51 ^St^ 5.67 ± 2.31 ^Sv^
*Aeromonas sobria*	5 10 15	**18.00 ± 1.73 ^Tx^** 16.00 ± 1.00 ^Tz^ 17.00 ± 0.00 ^Tb’^	5.67 ± 0.57 ^Uy^ **7.00 ± 1.73 ^UVa’^** 10.00 ± 1.73 ^Vc’^	**9.67 ± 3.51 ^Wy^** 9.00 ± 1.00 ^Wa’^ 9.67 ± 1.52 ^Wc’^
*Pseudomonas anguilliseptica*	5 10 15	**9.33 ± 2.89 ^Xd^**^’^ 10.00 ± 2.00 ^Xe’^ 11.33 ± 3.51 ^Xf’^	**10.67 ± 6.03 ^Yd’^** 11.00 ± 4.58 ^Ye’^ 12.33 ± 4.04 ^Yf’^	5.00 ± 2.00 ^Zd’^ **10.00 ± 1.00 ^A^**^’e’^ 13.67 ± 1.528 ^A’f’^
*Shewanella putrefaciens*	5 10 15	13.67 ± 1.15 ^B’g’^ 13.67 ± 0.57 ^B’i’^ **16.67 ± 1.528** ^C’k’^	**11.33 ± 2.08 **^D’g’h’^ 12.33 ± 3.06 ^D’i’^ 14.00 ± 3.61 ^D’k’l’^	**7.67 ± 2.52** ^E’h’^ 6.33 ± 1.52 ^E’j’^ 7.67 ± 3.06 ^E’l’^
*Vibrio cholerae*	5 10 15	5.33 ± 0.57 ^F’m’^ **6.67 ± 0.57** ^F’G’n’^ 8.33 ± 1.15 ^G’ñ’^	**5.33 ± 4.04 **^H’m’^ 7.33 ± 2.08 ^H’n’^ 8.33 ± 2.08 ^H’ñ’^	**1.333 ± 0.57** ^I’m’^ 3.33 ± 2.08 ^I’n’^ 4.00 ± 2.65 ^I’ñ’^
*Acinetobacter Iwoffii*	5 10 15	**8.33 ± 1.528** ^J’o’^ 10.00 ± 2.65 ^J’q’^ 11.67 ± 2.89 ^J’s’^	**8.67 ± 1.15 **^K’o’^ 9.67 ± 0.57 ^K’q’^ 11.67 ± 2.89 ^K’s’^	**1.60 ± 0.693** ^L’p’^ 1.30 ± 0.608 ^L’r’^ 0.93 ± 0.115 ^L’t’^
*Comamonas thiooxydans*	5 10 15	10.00 ± 0.00 ^M’u’^ **16.67 ± 0.577 **^N’w’^ 16.67 ± 2.08 ^N’y’^	6.67 ± 2.89 ^Ñ’u’v’^ **7.67 ± 1.528** ^Ñ’x’^ 9.67 ± 2.08 ^Ñ’z’^	3.00 ± 1.00 ^O’v’^ **8.00 ± 1.73** ^P’x’^ 9.67 ± 2.31 ^P’z’^
*Empedobacter falsenii*	5 10 15	**9.00 ± 0.00** ^Q’a”^ 10.00 ± 1.00 ^Q’c”d”^ 10.67 ± 1.528 ^Q’e”f^	**12.33 ± 2.52 **^R’a”^ 12.33 ± 2.08 ^R’c”^ 16.00 ± 4.36 ^R’e”^	**4.17 ± 2.02** ^S’b”^ 5.33 ± 3.21 ^S’d”^ 6.33 ± 3.06 ^S’f”^

Expressed values, expressed in mm, are the mean diameter of inhibition halos measured after being tested at different concentrations (15, 10, and 5 μL). Each species was evaluated separately. Uppercase letters express comparisons between EOs and bacterium individually; lowercase letters, and lowercase letters with apostrophes and quotation marks, express comparisons between bacterium and the EOs at the same vol. (µL). Bold numbers indicate the most effective dose at the lowest volume for each case; underlined numbers express the minimum dose with the higher inhibition halo compared in terms of the most effective dose per assay. Means that do not share a letter are significantly different (*p* < 0.05).

**Table 3:** Minimal Inhibitory Concentrations (MICs) of EOs (% *v*/*v*) determined by the agar microdilution method.

Strain	Cinnamon MIC	Thyme MIC	Tea Tree MIC
*Aeromonas* sp.	1	**<0.12**	2
*Aeromonas dhakensis*	**<0.12**	**<0.12**	2
*Aeromonas veronii*	1	**<0.12**	1
*Aeromonas veronii*	**<0.12**	**<0.12**	2
*Aeromonas veronii*	**<0.12**	**<0.12**	2
*Aeromonas sobria*	**<0.12**	**<0.12**	4
*Pseudomonas anguilliseptica*	0.5	**<0.12**	0.5
*Shewanella putrefaciens*	2	**<0.12**	2
*Comamonas thiooxydans*	1	**<0.12**	0.5
*Vibrio cholerae*	1	**<0.12**	2
*Acinetobacter Iwoffii*	1	**<0.12**	1
*Empedobacter falsenii*	0.25	**<0.12**	**>0.12**

These results are derived from the MICs of essential oils; bold numbers indicate the lowest concentration at which each strain was sensitive to the different EOs.

**Table 4:** Chemical composition of EOs.

Essential Oil	Chemical Composition
Cinnamon oil (*Cinnamomum zeylanicum*)	**trans-cinnamaldehyde (53.79%)** trans-cinnamyl acetate (9.83%) β-phellandrene (5.29%) β-caryophyllene (4.17%) linalool (3.01%) α-pinene (2.51%) para-cymene (2.33%) eugenol (2.12%) α-phellandrene (1.72%) limonene (1.51%) α-terpinene (1.37%) camphene (1.10%)
Tea tree (*Melaleuca alternifolia*)	**terpinen-4-ol (38.26%)** γ-terpinene (17.01%) α-terpinene (8.59%) α-terpineol (4.69%) terpinolene (3.15%) α-pinene (2.23%) delta-cadinene (2.22%) p-cymene (2.10%) 1,8-Cineole (1.97%) viridiflorene (1.90%) bicyclogermacrene (1.88%)
Thyme (*Thymus vulgaris*)	**Thymol (54.88%)** p-cymene (17.30%) carvacrol (3.33%) γ-terpinene (9.80%) Linalool (3.87%) myrcene (1.29%) β-caryophyllene (1.17%) borneol (1.13%) α-terpinene (1.12%)

Source: manufacturer’s datasheet. Bold text points to the main component of each EO.

**Table 5:** Bacterial strains previously isolated and accession numbers.

Microorganism	Accession Number
*Aeromonas* sp.	EF491849.1
*Aeromonas aquariorum*	FN796727.1
*Aeromonas veronii*	CP014774.1
*Aeromonas veronii*	JF490068.1
*Aeromonas veronii*	FR682763.1
*Aeromonas sobria*	AB526508.1
*Pseudomonas anguilliseptica*	MH185879.1
*Shewanella putrefaciens*	CP046329.1
*Vibrio cholerae*	CP026531.1
*Acinetobacter Iwoffii*	PP762073.1
*Comamonas thiooxydans*	AP026738.1
*Empedobacter falsenii*	MH712956.1

## Data Availability

The original contributions presented in this study are included in the article. Further inquiries can be directed to the corresponding author.

## Abbreviations

The following abbreviations are used in this manuscript:

EOs	Essential Oils
CEO	Cinnamon Essential Oil
TEO	Thyme Essential Oil
TTEO	Tea Tree Essential Oil
MAR Index	Multiple Antibiotic Resistance Index
DDt	Disc Diffusion Test
MICs	Minimal Inhibitory Concentrations
DMSO	Dimethyl Sulfoxide
